# Advancements and challenges in microalgal protein production: A sustainable alternative to conventional protein sources

**DOI:** 10.1186/s12934-025-02685-1

**Published:** 2025-03-10

**Authors:** Sameh S. Ali, Rania Al-Tohamy, Majid Al-Zahrani, Michael Schagerl, Michael Kornaros, Jianzhong Sun

**Affiliations:** 1 Biofuels Institute, School of the Environment and Safety Engineering, Jiangsu University, Zhenjiang, 212013 China; 2 Botany and Microbiology Department, Faculty of Science, Tanta University, Tanta, 31527 Egypt; 3 Biological Sciences Department, College of Science and Art at Rabigh, King Abdulaziz University, Rabigh, 25732 Saudi Arabia; 4 Department of Functional and Evolutionary Ecology, University of Vienna, Djerassiplatz 1, Vienna, 1030 Austria; 5 Laboratory of Biochemical Engineering & Environmental Technology (LBEET), Department of Chemical Engineering, University of Patras, Patras, 26504 Greece

**Keywords:** Microalgae, Sustainable protein, Food security, Bioavailability, Life cycle assessment, Biorefinery

## Abstract

The increasing global demand for sustainable protein sources necessitates the exploration of alternative solutions beyond traditional livestock and crop-based proteins. Microalgae present a promising alternative due to their high protein content, rapid biomass accumulation, and minimal land and water requirements. Furthermore, their ability to thrive on non-arable land and in wastewater systems enhances their sustainability and resource efficiency. Despite these advantages, scalability and economical feasibility remain major challenges in microalgal protein production. This review explores recent advancements in microalgal protein cultivation and extraction technologies, including pulsed electric field, ultrasound-assisted extraction, enzyme-assisted extraction, and microwave-assisted extraction. These innovative techniques have significantly improved protein extraction efficiency, purity, and sustainability, while addressing cell wall disruption and protein recovery challenges. Additionally, the review examines protein digestibility and bioavailability, particularly in the context of human nutrition and aquafeed applications. A critical analysis of life cycle assessment studies highlights the environmental footprint and economical feasibility of microalgal protein production compared to conventional protein sources. Although microalgal protein production requires significant energy inputs, advancements in biorefinery approaches, carbon dioxide sequestration, and industrial integration can help mitigate these limitations. Finally, this review outlines key challenges and future research directions, emphasizing the need for cost reduction strategies, genetic engineering for enhanced yields, and industrial-scale process optimization. By integrating innovative extraction techniques with biorefinery models, microalgal proteins hold immense potential as a sustainable, high-quality protein source for food, feed, and nutraceutical applications.

## Introduction

The global demand for protein is rapidly increasing due to population growth, rising incomes, and shifting dietary preferences [1]. By 2050, the global population is expected to grow by more than a third (approximately 2.3 billion), necessitating a 70% increase in food production [2]. Over the past half-century, advances in agricultural food production technologies and higher per capita income have significantly reduced worldwide hunger despite a doubling of the global population [3]. However, traditional protein sources, including animal-based products and conventional crops, present substantial environmental and economical challenges. These challenges encompass land degradation, excessive water use, and substantial greenhouse gas (GHG) emissions. Livestock farming, for instance, is a major contributor to deforestation, GHG emissions, and water consumption [4]. Similarly, large-scale cultivation of protein-rich crops demands extensive land and water resources, leading to habitat loss, soil degradation, and declining biodiversity [5]. The finite availability of arable land and freshwater further limits the scalability of conventional agriculture to meet the growing protein demand. Additionally, fluctuating costs of feed, fertilizers, and other inputs compromise the economical sustainability of traditional protein production systems [6]. Given these limitations, algal protein production emerges as a promising and sustainable solution to address global food security and environmental challenges.

Microalgae stand out as a transformative alternative due to their high protein content, rapid growth rates, and capacity to thrive in non-arable land and nutrient-rich wastewater systems [7]. Notably, microalgae are a rich source of high-quality proteins containing all essential amino acids. Species such as *Spirulina* and *Chlorella* boast protein contents of 50–70% and 40–60%, respectively, comparable to or exceeding conventional protein sources like soy and meat [8, 9]. Moreover, under optimal conditions, microalgae can double their biomass within hours, making them highly scalable [10]. Beyond protein, microalgae are rich in vitamins, minerals, and bioactive compounds, enhancing their nutritional profile and health benefits. A significant advantage of microalgae lies in their minimal resource requirements. They can be cultivated on non-arable land using saline water or wastewater, thereby reducing competition with food crops and alleviating freshwater use. Furthermore, microalgae play an active role in carbon capture, utilizing CO₂ during photosynthesis and enabling integration with industrial carbon capture systems to mitigate GHG emissions [11]. Microalgal biomass also offers economical value through co-products, such as lipids for biofuels, pigments for cosmetics, and antioxidants for nutraceuticals, fostering a multi-product biorefinery approach consistent with circular economy principles [12].

Despite these advantages, several critical challenges must be addressed to unlock the full potential of microalgal protein production. Improving microalgal growth rates and protein synthesis remains a priority, necessitating optimization of light exposure, nutrient availability, and cultivation conditions to maximize yields [13]. Efficient dewatering and processing techniques are also essential to enhance the economical viability of protein extraction. Innovations in harvesting technologies and biorefinery methods are actively being explored to overcome these bottlenecks [14]. However, the high costs associated with large-scale microalgal cultivation pose a significant barrier to widespread adoption. Cost-effective cultivation systems, coupled with value addition through co-products, are key to improving economical feasibility [15]. Additionally, comprehensive life cycle assessments (LCAs) are crucial to evaluate the environmental impacts of microalgal protein production. These assessments must consider energy consumption, GHG emissions, and resource utilization throughout the production process to ensure sustainability [16]. Regulatory frameworks and safety standards are equally important for building consumer trust and facilitating the integration of microalgal proteins into mainstream food markets [17]. By addressing these challenges and leveraging the unique attributes of microalgae, microalgal protein production has the potential to become a sustainable and economically viable alternative to conventional protein sources. It can significantly contribute to global food security while reducing environmental impacts.

The objective of this review is to provide a comprehensive analysis of the latest advancements, challenges, and future perspectives in microalgal protein production. Specifically, this review aims to (i) assess microalgal cultivation methods and their role in optimizing protein yields while minimizing environmental impacts; (ii) examine innovative protein extraction techniques, including pulsed electric field (PEF), ultrasound-assisted extraction (UAE), enzyme-assisted extraction (EAE), and microwave-assisted extraction (MAE), focusing on their efficiency, sustainability, and scalability; (iii) evaluate the digestibility and bioavailability of microalgal proteins for human nutrition and aquaculture applications; (iv) analyze the environmental and economical viability of microalgal protein production through LCAs; and (v) identify key challenges and propose future directions for improving the cost-effectiveness, scalability, and industrial adoption of microalgal protein technologies. By addressing these aspects, this review aims to provide valuable insights for researchers, policymakers, and industry stakeholders to promote the integration of microalgal proteins as a sustainable alternative in the global food and feed industries.

### Microalgal cultivation

Microalgae are photosynthetic microorganisms that have gained significant attention as a sustainable source of protein, biofuels, and high-value compounds [18]. Microalgal cultivation is a cornerstone of the biotechnology industry, offering pathways for sustainable biomass production and the development of valuable bio-based products. Microalgal cultivation can be classified into four primary metabolic pathways: phototrophic, heterotrophic, mixotrophic, and photoheterotrophic. Each of these approaches provides unique advantages tailored to specific industrial applications, as summarized in Table 1 [19–25]. Each pathway offers unique advantages and challenges, influencing their suitability for large-scale production. This section provides a detailed analysis of these cultivation methods, supported by recent studies, and highlights their environmental and economical implications.

**Table 1:** Comparison of microalgal cultivation methods for protein production

Cultivation method	Energy source	Carbon source	Applications	Advantages	Challenges	Suitability for large-scale production	References
Phototrophic	Light	CO₂	• Biofuels • Food • Bioremediation	• Sustainable • Scalable, • Low-cost • Mitigates CO₂ emissions	• Light dependency • Low cell density • Contamination risks • High evaporation rates	High	[19, 20]
Heterotrophic	Organic compounds	Organic compounds	• Biofuels • Bioplastics • Nutraceuticals	• High biomass productivity • Controlled growth • Flexible substrate use	• Expensive substrates • Contamination risks	Moderate	[21, 22]
Mixotrophic	Light & organics	CO₂ & organic compounds	• Biofuels • High-value products	• Versatility • Enhanced biomass and metabolite production • CO₂ recycling	• Cost of organics • Metabolic complexity	Low	[23, 24]
Photoheterotrophic	Light	Organic compounds	• Specialty metabolites • Biopharmaceuticals	• Enhanced light- metabolite production	• High costs • Limited scalability	Low	[22, 25]

### Phototrophic cultivation

Phototrophic cultivation is the most widely used method for microalgal production, leveraging sunlight as the primary energy source and CO₂ as the carbon source [19]. This approach is highly scalable and suitable for outdoor biomass production, making it a preferred choice for large-scale operations. Phototrophic cultivation is particularly advantageous for its ability to mitigate industrial CO₂ emissions by integrating microalgal systems with flue gas from power plants or industrial facilities [26]. Light is a critical factor in phototrophic cultivation, directly affecting biomass productivity and protein biosynthesis. Optimal light conditions vary among species, with higher light intensities generally leading to increased biomass production. For instance, Nzayisenga et al. [27] demonstrated that light intensities of 50, 150, and 300 µE/m²/s significantly influenced biomass and fatty acid production in *Desmodesmus* and *Scenedesmus obliquus*. Higher light intensities increased biomass but reduced protein content, highlighting the need for species-specific optimization. Besides light, microalgae act as carbon sinks, sequestering CO₂ during photosynthesis. Integrating microalgal cultivation with industrial CO₂ sources can enhance sustainability and reduce GHG emissions. For example, *Arthrospira platensis* cultivated under high light intensity (2300 µmol/m²/s) achieved a biomass productivity of 0.62 g/L/d, energy consumption efficiency of approximately 2.26–2.31 g/kWh/d, and a photosynthetic efficiency of 8.02% [28]. Despite its advantages, phototrophic cultivation faces challenges such as light limitation in dense cultures, contamination risks in open systems, and high evaporation rates in outdoor ponds [20]. To address these limitations, innovations in photobioreactor design and light manipulation are being explored. Closed photobioreactors (PBRs) offer better control over growth conditions but are energy-intensive and costly to operate [10].

**Fig. 1:**
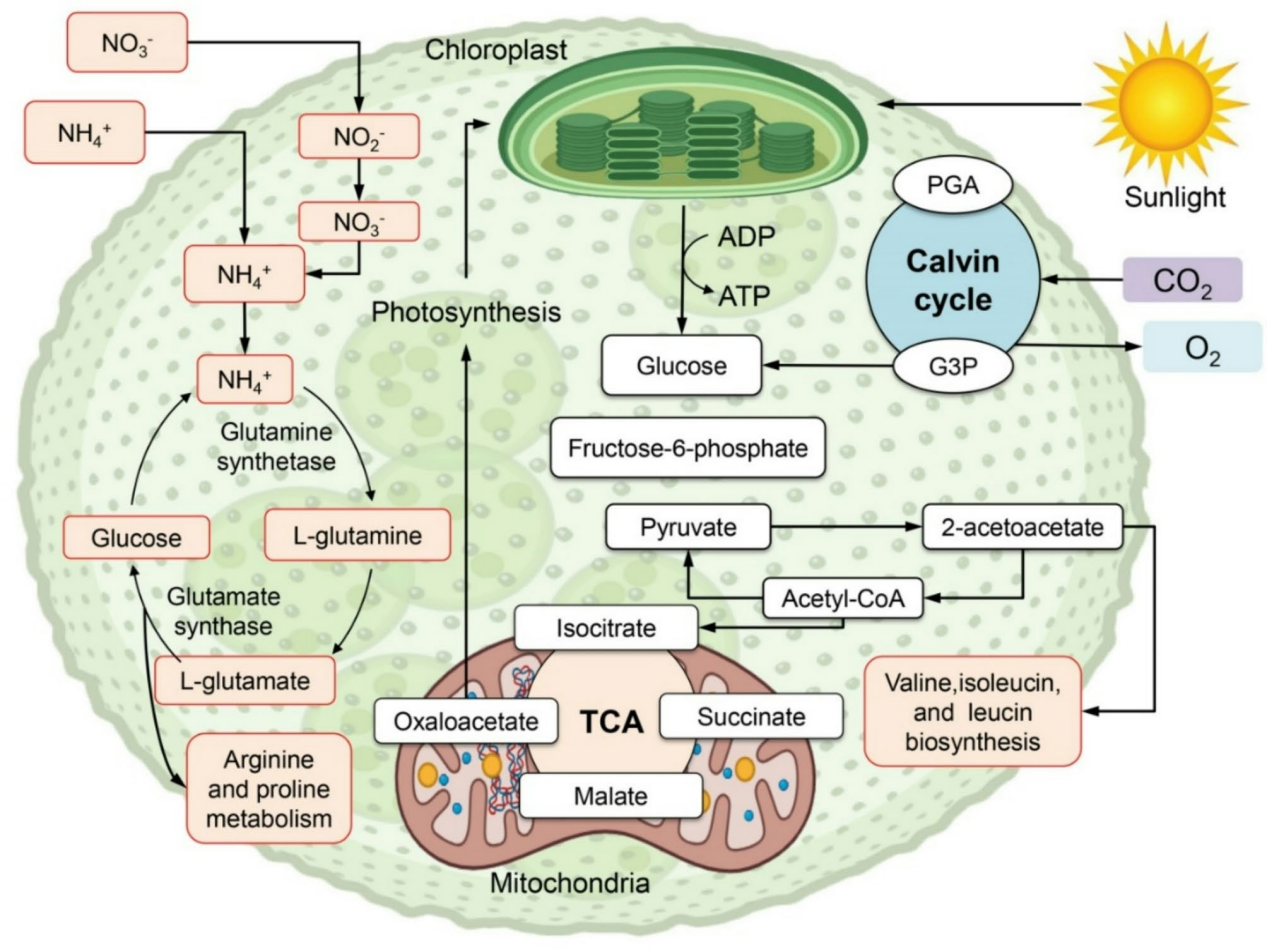
Metabolic pathways involved in microalgal phototrophic cultivation, highlighting the key processes occurring in the chloroplast and mitochondria

The metabolic pathways involved in microalgal phototrophic cultivation (Fig. 1) illustrate key processes in the chloroplast and mitochondria. During photosynthesis in the chloroplast, sunlight serves as the primary energy source, driving the Calvin cycle to convert CO₂ into glyceraldehyde-3-phosphate (G3P) and glucose, transforming light energy into chemical energy [29]. This cycle relies on ATP and NADPH generated during light-dependent reactions. Nitrogen metabolism further supports microalgal growth by assimilating nitrate (NO₃⁻) or ammonium (NH₄⁺) into amino acids like glutamine and glutamate via glutamine synthetase and glutamate synthase. These amino acids are vital for synthesizing proteins and nitrogen-containing biomolecules, including those involved in arginine and proline metabolism. Glucose from photosynthesis undergoes glycolysis, producing pyruvate, which enters the mitochondrial tricarboxylic acid (TCA) cycle. The TCA cycle generates energy and provides intermediates like oxaloacetate and 2-acetoacetate, which are precursors for essential amino acids such as valine, isoleucine, and leucine. This integration of photosynthetic carbon fixation with cellular metabolism underpins biomass growth and protein production [30]. Additionally, photosynthesis releases oxygen (O₂) as a byproduct during water splitting in the light-dependent reactions.

### Heterotrophic cultivation

Heterotrophic cultivation involves growing microalgae in the absence of light by utilizing organic carbon sources such as glucose or acetate. This method is particularly effective for achieving high cell densities and producing high-value compounds like lipids and pigments. Compared to phototrophic methods, heterotrophic cultivation can yield significantly higher biomass. For instance, *Chlorella vulgaris* cultivated heterotrophically demonstrated a 4.5-fold reduction in environmental impact when optimized with hydrolyzed food waste [31]. Additionally, organic waste streams, such as food waste or agricultural by-products, can serve as substrates, reducing production costs and enhancing sustainability [32–33]. However, heterotrophic cultivation faces several challenges, including high costs and contamination risks. The expense of organic substrates and energy-intensive processes makes this method economically demanding. Heterotrophic cultivation costs can be up to 80% higher than phototrophic methods [22]. Furthermore, heterotrophic systems are susceptible to bacterial contamination, especially in open systems, which necessitates stringent sterilization measures [34].

### Mixotrophic cultivation

Mixotrophic cultivation combines phototrophic and heterotrophic metabolism, enabling microalgae to utilize both light and organic carbon sources simultaneously [24]. This approach offers flexibility and can enhance biomass productivity and metabolite synthesis. The advantages of mixotrophic cultivation include increased biomass and metabolic production, as well as improved CO₂ recycling [23]. Studies have shown that mixotrophic cultivation can significantly boost biomass productivity and the synthesis of high-value compounds. For example, *Chlorella vulgaris* cultivated under mixotrophic conditions demonstrated higher cell productivity compared to purely phototrophic or heterotrophic conditions [35]. Additionally, *Chlorella vulgaris* cultured mixotrophically with acetate supplementation achieved biomass yields 6.8 times higher than those achieved through autotrophic cultivation alone [22]. The CO₂ released during respiration is reused in photosynthesis, enhancing carbon efficiency and reducing emissions [36]. This method supports CO₂ recycling, aligning with circular economy principles. However, mixotrophic cultivation faces challenges that limit its widespread application. The high costs of organic substrates and the need for both light and organic carbon sources increase operational expenses, making large-scale production economically challenging [37]. Additionally, maintaining optimal conditions for both phototrophic and heterotrophic metabolism requires advanced bioreactor designs and precise process control systems [38].

### Photoheterotrophic cultivation

Photoheterotrophic cultivation involves the use of organic carbon sources in combination with light energy to enhance metabolite production. While this method holds promise for producing specialty biopharmaceuticals, its high operational costs and technical complexity limit scalability [25]. A key advantage of photoheterotrophic conditions is their ability to increase the production of specific metabolites, such as carotenoids and phycobiliproteins, which are highly valuable in the nutraceutical and cosmetic industries [39]. However, the requirement for both organic substrates and light, along with the need for specialized bioreactor designs, makes this method economically unfeasible for large-scale production [40]. Overall, microalgal cultivation provides a sustainable and versatile platform for protein production, with each metabolic pathway offering unique advantages and challenges. Phototrophic cultivation remains the most viable option for large-scale production due to its low cost and scalability. However, advancements in heterotrophic and mixotrophic methods, combined with technological innovations and policy support, could further enhance the economical and environmental viability of microalgal protein production. By addressing current challenges and leveraging the unique advantages of microalgae, this field has immense potential to contribute to global food security and sustainability. Therefore, future research should focus on optimizing cultivation systems, improving strain resilience, and reducing costs through innovative bioprocess engineering and LCAs.

## Harvesting in microalgal protein production

Harvesting is a critical step in microalgal protein production, involving the separation of microalgal biomass from the culture medium [41]. This process is often energy-intensive and constitutes a significant portion of overall production costs. Efficient harvesting methods are essential for maximizing biomass recovery, reducing energy consumption, and preserving protein quality. This section provides an in-depth analysis of microalgal harvesting techniques, their advantages, challenges, and implications for protein production. One of the primary challenges in harvesting microalgae is their low biomass concentration, which makes the process both costly and energy-intensive [42]. The selection of an appropriate harvesting method depends on various factors, including microalgal species, cell size, culture medium composition, and intended application. Since microalgae are microscopic and dispersed in water, their separation from the culture medium requires specialized techniques.

The main harvesting methods include sedimentation, centrifugation, filtration, flotation, and flocculation (Table 2). Sedimentation is a gravity-based technique in which algal cells gradually settle at the bottom of a tank. While it is a low-cost method, it is slow and inefficient for species with low settling velocities. Its efficiency can be improved by modifying environmental conditions such as pH and temperature [43]. In contrast, centrifugation employs centrifugal force to rapidly separate microalgal biomass from the medium. Although highly effective, this method is energy-intensive, making it less suitable for large-scale applications. Industrial-scale centrifuges can process large volumes efficiently but require substantial capital investment [44]. Filtration involves passing the algal culture through membranes or filter media, which retain algal cells while allowing water to pass through [45]. This technique is effective for large-celled or filamentous algae but is prone to clogging in dense cultures. Advances in membrane technology, such as ultrafiltration and microfiltration, have enhanced filtration efficiency. Flotation relies on air bubbles attaching to algal cells, causing them to float to the surface for easy collection. While effective, this method often requires chemical surfactants. Dissolved air flotation (DAF) is a widely used flotation technique that improves recovery efficiency by introducing microbubbles to enhance cell separation [46]. Flocculation involves adding chemical or biological agents that aggregate microalgal cells into larger flocs, which can be easily separated [47]. This method is cost-effective but may introduce impurities that affect protein extraction. To address concerns about contamination, natural and bio-based flocculants are being developed.

**Table 2:** Comparison of microalgal harvesting techniques for protein production

Method	Efficiency	Energy demand	Cost	Suitability for large-scale production	References
Sedimentation	Low	Low	Low	Suitable for dense cultures with high settling velocity	[43]
Centrifugation	High	High	High	Suitable for high-value applications	[44]
Filtration	Moderate	Moderate	Medium	Effective for large-cell algae but prone to clogging	[45]
Flotation	Moderate	Moderate	Medium	Effective but requires chemical additives	[46]
Flocculation	High	Low to Moderate	Low	Cost-effective but may introduce impurities	[47]

The efficiency, energy consumption, and cost implications of different harvesting methods are compared in Table 2. Efficient harvesting plays a crucial role in protein extraction, as poorly harvested biomass can lead to losses and contamination. After harvesting, microalgae undergo drying and cell disruption before protein extraction. Common drying methods include spray drying, freeze drying, and oven drying, each with limitations regarding protein preservation. The efficiency of protein extraction is closely linked to the harvesting method, as residual chemicals from certain techniques may compromise protein purity. While advancements in harvesting technologies continue, challenges related to energy efficiency, cost reduction, and scalability persist [48]. Hybrid methods, which combine multiple harvesting techniques, are being explored to enhance efficiency. Additionally, genetic and metabolic engineering of microalgae may improve natural aggregation properties, facilitating easier harvesting. Currently, harvesting accounts for 20–30% of the total production cost in microalgal protein production [49], making energy and cost reduction critical for improving economical viability. Moreover, the environmental impact of harvesting methods, particularly those involving chemical flocculants, must be carefully evaluated to ensure sustainability. Future research should focus on developing cost-effective and energy-efficient harvesting methods suitable for large-scale operations. Integrating harvesting with downstream processing steps, such as cell disruption and protein extraction, can streamline production and reduce costs. Environmentally friendly harvesting approaches, including bioflocculation and autoflocculation, offer promising solutions for improving sustainability [50].

In conclusion, harvesting is a vital step in microalgal protein production, significantly influencing overall yield and cost-effectiveness. While various methods exist, selecting the most suitable technique depends on factors such as energy efficiency, cost, and scalability. Continued research and optimization of harvesting methods will play a key role in establishing microalgae as a viable and sustainable protein source.

## Genetic engineering for enhanced microalgal harvesting in protein production

Genetic engineering has emerged as a promising approach to enhancing the efficiency of microalgal harvesting for protein production [51]. One of the primary challenges in microalgal harvesting is the low biomass concentration and the high energy demand required for separation from the culture medium. Genetic modifications can address these challenges by improving cell aggregation properties, altering surface charge, and modifying extracellular polymeric substances (EPS) to facilitate easier harvesting [52].

**Fig. 2:**
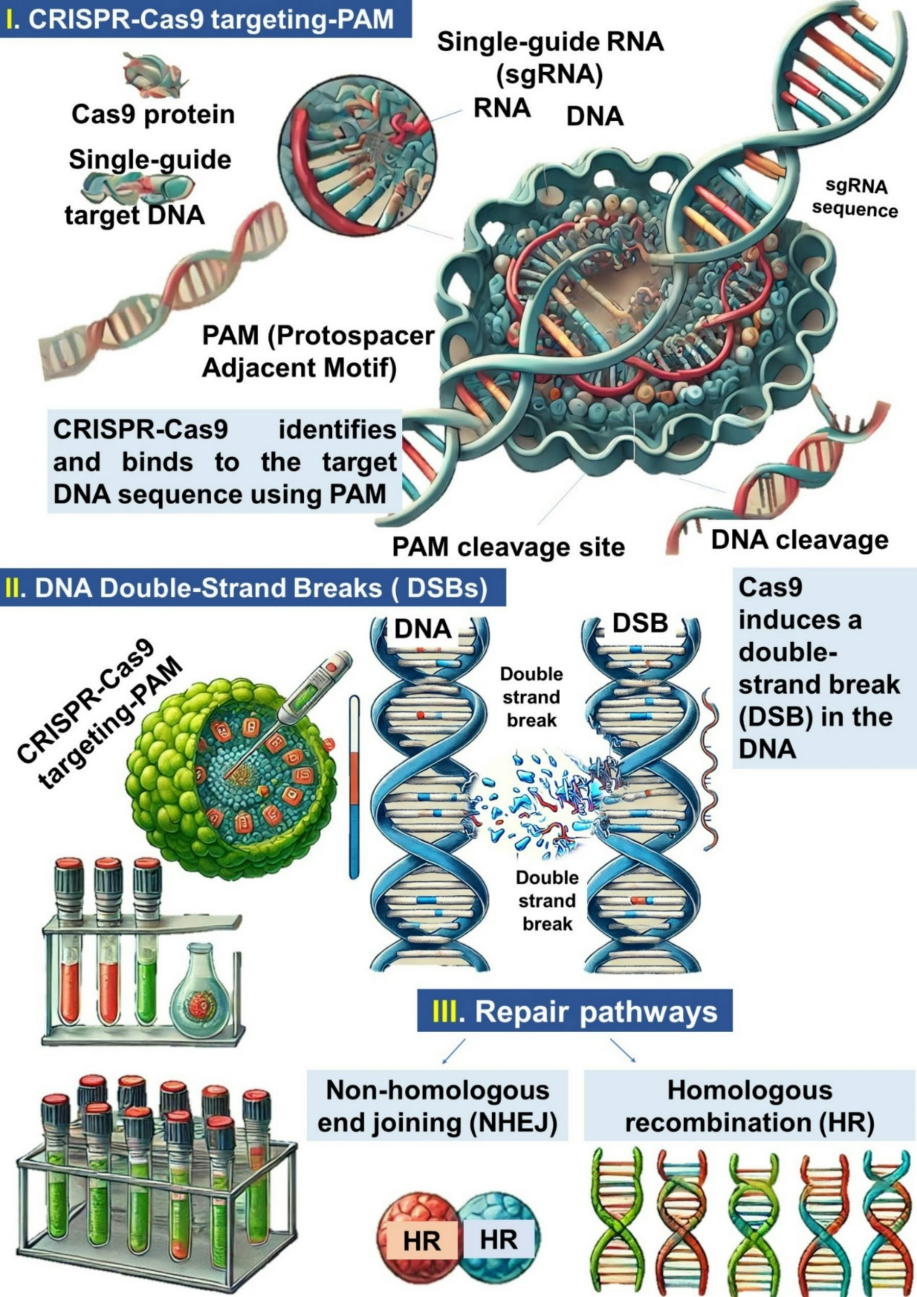
CRISPR-Cas9 for microalgal protein production. CRISPR-cas9 targeting- protospacer adjacent motif (PAM), DNA double-strand breaks (DSBs), and repair pathways

**Fig. 3:**
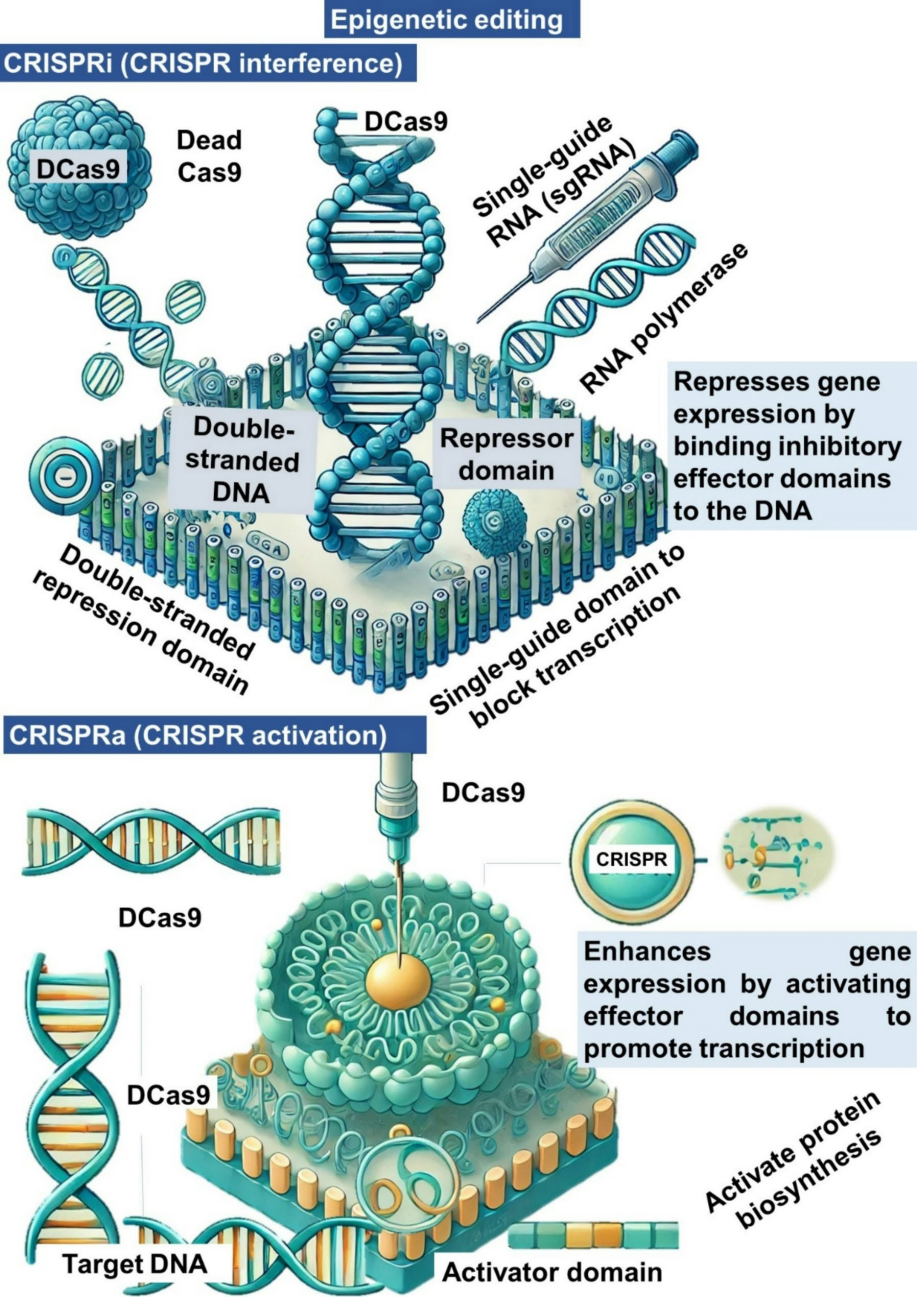
Epigenetic editing for microalgal protein production. CRISPR interference (CRISPRi) and CRISPR activation (CRISPRa)

CRISPR-Cas9 technology has proven to be a powerful tool for precise and targeted genome editing in microalgae (Figs. 2 and 3). The Cas9 protein, guided by a single-guide RNA (sgRNA), binds to the target genomic DNA by recognizing a protospacer adjacent motif (PAM) sequence, as illustrated in Fig. 2. Cas9 induces a double-strand break (DSB) in the DNA, which can be repaired through two pathways: (i) non-homologous end joining (NHEJ), an error-prone repair mechanism that introduces small insertions or deletions (indels), disrupting target genes to improve microalgal metabolic efficiency for protein synthesis; and (ii) homologous recombination (HR), a precise repair mechanism that uses a donor DNA template for targeted knock-in or knock-out modifications. This approach can be applied to enhance genes associated with protein production in microalgae. As shown in Fig. 3, the dCas9 protein, a nuclease-deactivated version of Cas9, is fused with effector domains to regulate gene expression epigenetically. In CRISPRi (CRISPR interference), inhibitory effector domains repress gene transcription, potentially suppressing competing pathways and redirecting metabolic resources toward protein synthesis. Conversely, in CRISPRa (CRISPR activation), activator effector domains enhance the expression of target genes, thereby promoting protein biosynthesis and improving the yield and quality of microalgal proteins. Zhang et al. [53] reported that the CRISPR-Cas9 system is predominantly employed in five approaches: insertion, deletion, knock-out, and knock-in strategies, which directly alter chromosomal DNA, as well as interference strategies that disrupt mRNA transcription while maintaining the original DNA sequence. By modifying genes involved in cell adhesion, flocculation, and biofilm formation, researchers can engineer algal strains that naturally aggregate, reducing the need for energy-intensive harvesting methods such as centrifugation and filtration. For instance, targeted disruption of genes regulating surface hydrophobicity or the secretion of adhesion-promoting biomolecules can enhance auto-flocculation, simplifying biomass recovery. Efforts are also underway to fine-tune CRISPR-Cas9 strategies to minimize off-target effects, ensuring stable and predictable modifications that improve harvesting efficiency.

Synthetic biology further strengthens genetic engineering strategies by enabling the design of custom genetic circuits that regulate cell surface properties. Researchers can introduce regulatory elements that trigger flocculant production in response to environmental signals, such as nutrient depletion or changes in pH, thereby enabling controlled and efficient harvesting [54, 55]. Additionally, metabolic engineering can be employed to upregulate the production of natural bio-flocculants, reducing the need for chemical additives that could compromise protein purity. Another promising approach involves modifying algal cell walls to improve sedimentation and flotation properties. By manipulating genes involved in EPS production, researchers can enhance the secretion of sticky polysaccharides that promote cell aggregation [56]. This strategy has been explored in species such as *Chlamydomonas reinhardtii* and *Dunaliella salina*, where engineered mutants exhibit improved settling rates, significantly reducing the energy required for biomass recovery [57]. Furthermore, genetic modifications can optimize the secretion of exopolysaccharides that enhance biofilm formation, facilitating the development of biofilm-based harvesting systems [58]. Engineered strains that form structured biofilms on solid surfaces enable continuous, low-energy harvesting strategies, making them particularly attractive for large-scale protein production.

Despite these advancements, challenges remain in ensuring stable transgene expression and avoiding unintended metabolic burdens that may impact overall biomass productivity. Future research should focus on refining gene-editing techniques, improving the stability of engineered traits, and integrating genetic modifications with scalable harvesting technologies. As genetic engineering tools continue to evolve, they hold great potential for transforming microalgal harvesting, ultimately enhancing the economical viability and sustainability of microalgal protein production.

## Large-scale extraction approaches of protein from microalgae

Microalgae are a promising source of sustainable proteins for applications in food, feed, and pharmaceuticals. However, extracting proteins from microalgae at an industrial scale remains challenging due to their resilient cell walls and the intracellular localization of proteins [59]. Recent advancements in extraction methods have focused on enhancing cell disruption, maximizing protein yield, and minimizing energy consumption. Table 3 presents a comparison of advanced microalgal protein extraction techniques alongside conventional methods [60–75].

**Table 3:** Comparison of advanced microalgal protein extraction techniques alongside conventional methods

Method	Principle	Advantages	Limitations	Sustainability for large-scale use	References
**Conventional methods**					
Mechanical disruption (bead milling & homogenization)	• Physically breaks cell walls	• Effective for tough microalgae	• High energy cost • Heat-induced protein degradation	High	[60, 61]
Chemical extraction (solvent, acid, and alkali)	• Dissolves cell walls using chemicals	• High protein yield	• Harsh chemicals can denature proteins and be environmentally hazardous	Medium	[62, 63]
Sonication	• Uses sound waves to create cavitation	• Effective for small-scale applications	• Can damage proteins with prolonged exposure	Low	[64, 65]
**Advanced methods**					
Enzyme-assisted extraction	• Uses specific enzymes to degrade the cell wall	• Low energy • High protein purity • Eco-friendly	• Expensive enzymes • Species-specific efficiency	Medium-High	[66, 67]
Bead milling with centrifugation	• Mechanical grinding followed by separation	• Scalable • Effective cell disruption	• High energy consumption	High	[68, 69]
Ultrasound-assisted extraction	• High-frequency ultrasound for cavitation	• Fast • Efficient • Minimal solvent use	• Risk of protein degradation	Medium	[70, 71]
Pulsed electric field	• High-voltage pulses create pores in cell walls	• Low energy • Preserves protein structure	• High initial cost	High	[72, 73]
Supercritical fluid extraction	• Uses supercritical CO₂ and co-solvents	• High purity • No toxic solvents	• Expensive • Requires technical expertise	Medium	[74, 75]

### Pulsed electric field

**Fig. 4:**
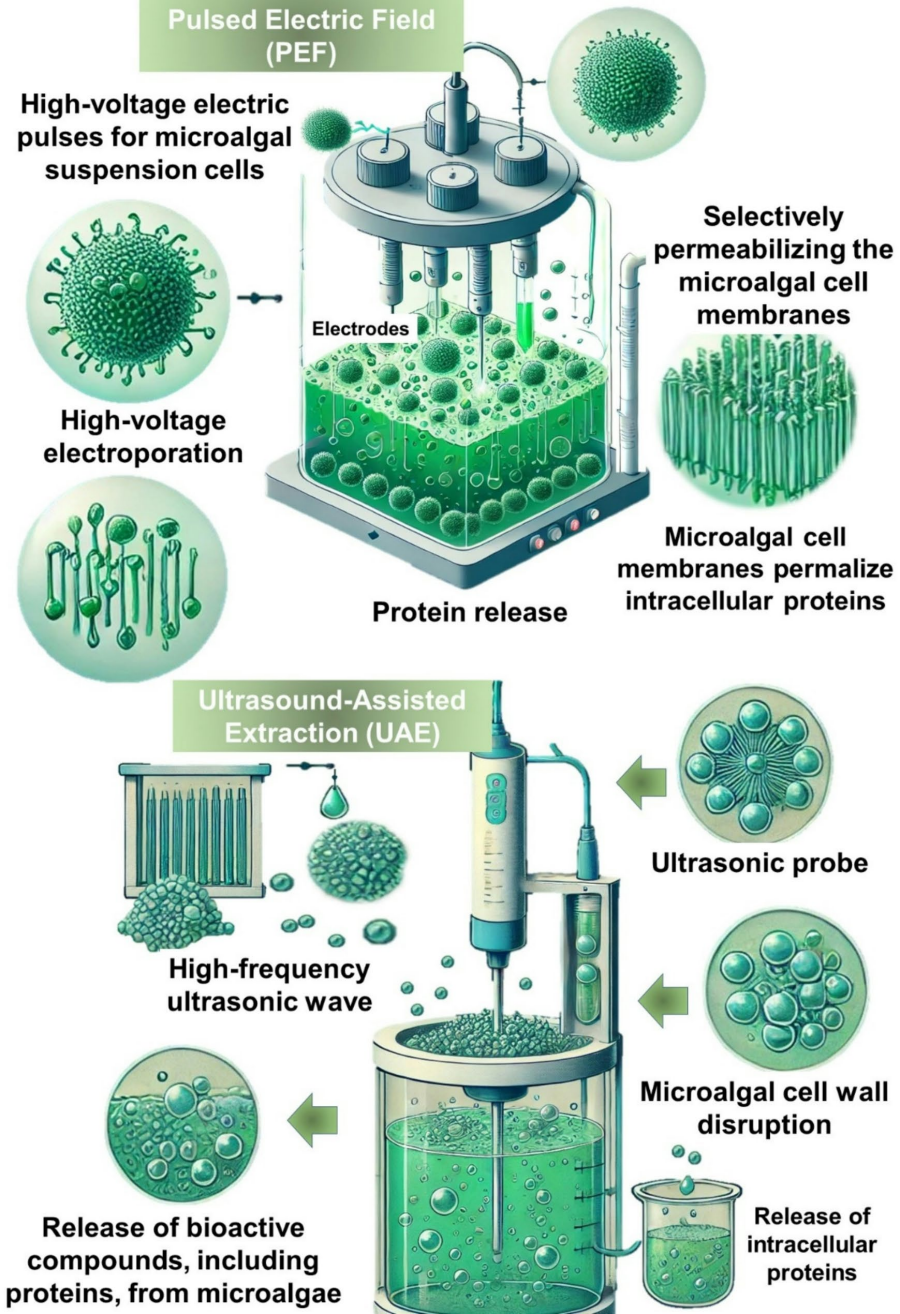
Pulsed electric field (PEF) and ultrasound-assisted extraction (UAE) technologies for protein extraction from microalgal cells

Pulsed Electric Field (PEF) treatment has emerged as a promising and environmentally friendly method for inactivating microalgae and facilitating the release of intracellular compounds [72]. This technique utilizes short, high-voltage electric pulses—typically lasting from microseconds to milliseconds—applied between two electrodes to enhance the permeability of microalgal cell membranes through a process known as electroporation (Fig. 4). By selectively permeabilizing cell membranes, PEF enables the extraction of intracellular biomolecules, including proteins, pigments, and lipids, making it a valuable tool for applications in food, feed, biofuels, nutraceuticals, and other high-value industries [73].

PEF technology operates by applying an electric field that disrupts the integrity of microalgal cell membranes, inducing pore formation. The process is energy-efficient, scalable, and preserves the structural integrity of extracted proteins [76]. A major advantage of PEF extraction is its cost-effectiveness, as it primarily uses water as the pulsing medium, eliminating the need for hazardous solvents or harsh chemicals [77]. Furthermore, the method is compatible with post-pulse buffers, allowing for seamless integration with downstream processing techniques. Despite its advantages, the efficacy of PEF treatment is influenced by multiple factors, including microalgal cell size, electric field strength, pulse duration, and electrode configuration [78]. Smaller microalgae cells may require higher electric field strengths to achieve comparable effects, potentially increasing energy consumption [79]. Additionally, challenges in scaling up PEF technology include optimizing electrode gap and conductivity to ensure uniform electric field distribution across larger volumes.

The effectiveness of PEF extraction compared to conventional methods has been demonstrated in several studies. Parniakov et al. [80] investigated the potential of PEF pre-treatment as an initial step in pH-assisted aqueous extraction of microalgal components from *Nannochloropsis* suspensions. The study compared PEF pre-treatment with sonication under normal (pH = 8.5) and basic (pH = 11) conditions. The findings revealed that PEF enabled the selective extraction of a fraction of pure proteins distinct from those obtained through sonication-pretreated suspensions. These results highlight the advantage of PEF pre-treatment under normal conditions and its potential for supplementary extraction under alkaline conditions.

Despite its promise, the widespread adoption of PEF extraction in microalgal biorefineries requires further optimization of processing parameters such as pulse duration, frequency, and electric field strength [81]. These factors play a critical role in maximizing extraction efficiency while minimizing potential adverse effects on cellular integrity and product quality. Moreover, the scalability and cost-effectiveness of PEF extraction systems must be thoroughly assessed to facilitate commercial implementation [14]. Future research should focus on optimizing PEF treatment conditions for different microalgal species, exploring its integration with other extraction techniques (e.g., enzymatic or ultrasound-assisted extraction), and evaluating its long-term energy requirements and environmental impact in industrial settings. With continued advancements, PEF technology has the potential to revolutionize microalgal bioprocessing by providing a sustainable and efficient method for intracellular compound extraction, aligning with the increasing demand for green biotechnologies.

### Ultrasound-assisted extraction

Ultrasound-assisted extraction (UAE) is an advanced technique that employs high-frequency ultrasonic waves to enhance the extraction of bioactive compounds, including proteins, from microalgae [70]. This process generates cavitation—a phenomenon where bubbles in the liquid medium rapidly collapse due to alternating low- and high-pressure cycles induced by ultrasonic waves—leading to microalgal cell wall disruption and the subsequent release of intracellular proteins and other biomolecules [71] as shown in Fig. 4. Due to its efficiency, eco-friendliness, and scalability potential, UAE has gained significant attention as a viable alternative to conventional extraction methods. Moreover, UAE can be integrated with enzymatic or solvent-assisted extraction to enhance protein yield and overall extraction efficiency [82]. However, the method requires optimization for different microalgal species, as prolonged ultrasonic exposure may lead to protein degradation.

UAE is characterized by its ability to enhance internal diffusion, promote the formation of eddies, and improve the mass transfer of solutes from the intracellular environment to the solvent medium [83]. These mechanical effects lead to greater disruption of algal cell structures, facilitating the release of intracellular proteins and other valuable compounds. A study by Gayathri et al. [84] demonstrated that the propagation of sound waves creates cavitation at regular intervals, effectively rupturing microalgal cells. Additionally, UAE has been recognized as an energy-efficient and environmentally sustainable extraction technique, as it eliminates the need for hazardous chemicals and reduces overall processing time.

Numerous studies have evaluated the efficacy of UAE in protein extraction and its impact on digestibility, solubility, and bioavailability. In this context, Janczyk et al. [85] investigated the effects of ultrasound treatment on *Chlorella vulgaris* protein digestibility in rats. The study found that ultrasound treatment significantly increased crude protein digestibility (56.7%) compared to electroporation (44.3%) and untreated spray-dried *Chlorella vulgaris* (46.9%). Additionally, the ultrasound-treated proteins exhibited improved protein efficiency ratios and nitrogen balance, suggesting enhanced nutritional benefits. Rodrigues et al. [86] employed UAE for the recovery of phycobiliproteins from *Spirulina platensis* and *Arthrospira platensis* using protic ionic liquids as solvents. A central rotational composite design was used to optimize the solvent-to-biomass ratio and pH conditions. The study found that the highest concentrations of phycobiliproteins were obtained using a 2-hydroxyethylammonium acetate (2-HEAA) and 2-hydroxyethylammonium formate (2-HEAF) solvent mixture at pH 6.5, with a solvent-to-biomass ratio of 7.9 mL/g and an extraction duration of 30 min. Among the extracted pigments, allophycocyanin was the most abundant (6.3 mg/g), followed by phycocyanin (5.95 mg/g) and phycoerythrin (2.6 mg/g), demonstrating the effectiveness of UAE in pigment recovery. Liu et al. [87] investigated the combination of enzymatic pretreatment using Viscozyme followed by UAE for the extraction of lipids and proteins from *Nannochloropsis oleoabundans*. The combined process achieved a higher degree of cellular disruption and lipid recovery than UAE alone, highlighting UAE’s potential for enhancing the extraction efficiency of multiple bioactive compounds from microalgae. Additionally, UAE applied after enzymatic pretreatment significantly increased lutein yield from *Chlorella vulgaris*, reaching 3.36 mg/g—higher than UAE alone (3.16 mg/g). Lee et al. [88] applied UAE to extract proteins from *Chlorella vulgaris* using ionic liquid-based aqueous solutions. The study compared the efficiency of UAE using Good’s buffer ionic liquids with conventional extraction buffers by assessing cell structure disruption. The optimized UAE process, which utilized 6 g biomass/L, [Ch][MOPSO]-HCl buffer (50 mM), an ultrasound exposure time of 30 min, and an ultrasound power of 400 W, yielded a protein content of 25.3% dry weight (Dw). UAE achieved significantly higher protein yields compared to other methods, such as freeze-thawing and non-ionic detergent treatments (e.g., Triton X-100) [89].

While UAE offers significant advantages in protein extraction from microalgae, several challenges must be addressed for its large-scale industrial application. The efficiency of UAE depends on various factors, including microalgal species, cell wall composition, solvent choice, ultrasound power, duration, and frequency [90]. Optimizing these parameters is crucial for maximizing protein yield while minimizing structural degradation. Additionally, prolonged ultrasonic exposure can cause localized heating and free radical generation, potentially leading to protein denaturation or degradation [91]. Therefore, precise control of process conditions is essential to maintain protein integrity. While UAE is considered energy-efficient at a laboratory scale, its feasibility for large-scale industrial applications requires further investigation. Key challenges include transducer design, energy consumption, and the selection between batch and continuous flow processing systems. Moreover, integrating UAE with enzymatic, chemical, or PEF techniques could further enhance protein extraction efficiency while mitigating process limitations [92]. Continued research and process optimization will be critical in advancing UAE as a scalable and sustainable method for microalgal protein extraction.

### Enzyme-assisted extraction

**Fig. 5:**
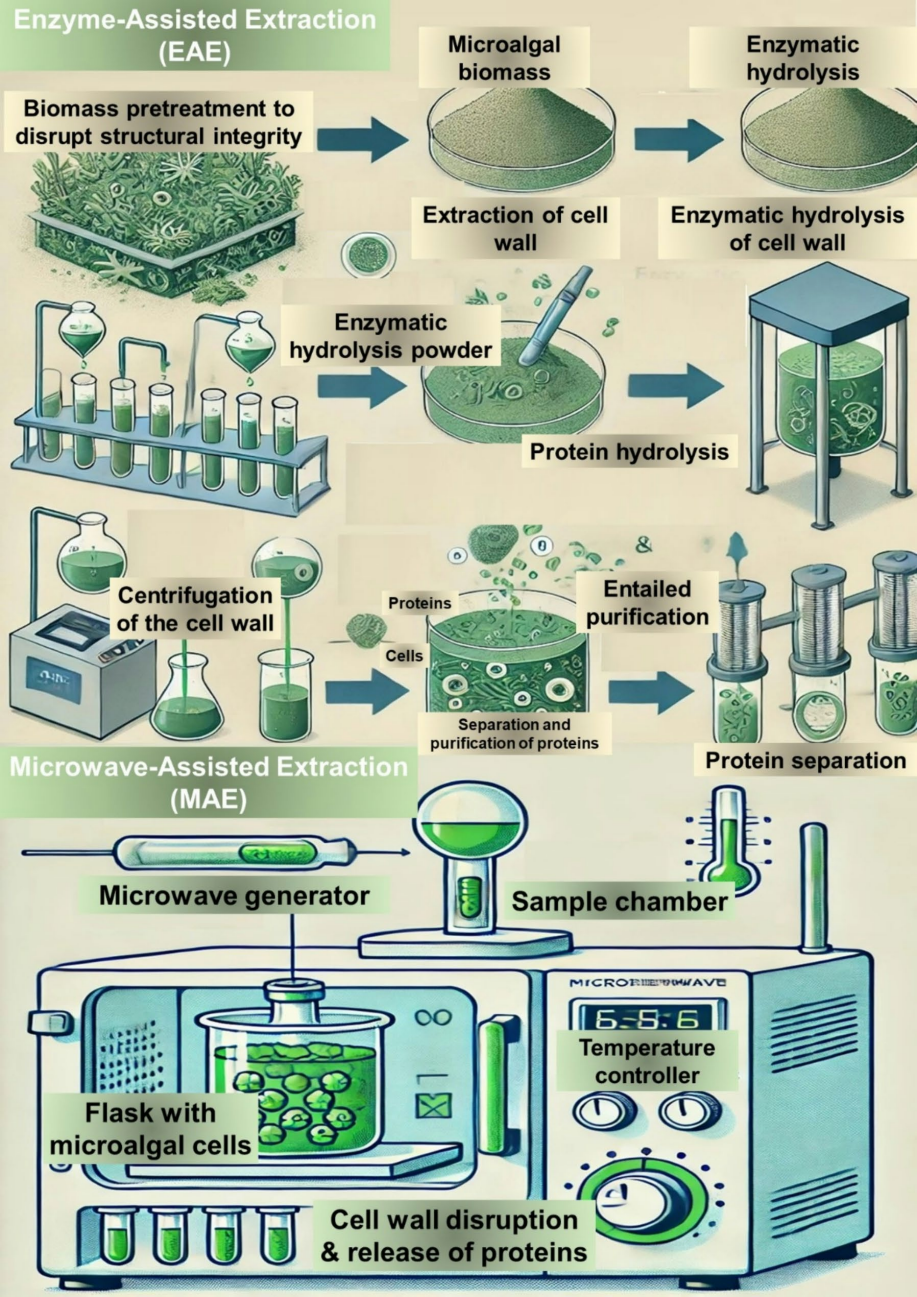
Enzyme-assisted extraction (EAE) and microwave-assisted extraction (MAE) technologies for protein extraction from microalgal cells

Enzyme-assisted extraction (EAE) is an emerging biotechnological approach for recovering proteins from microalgal biomass [66]. This method employs specific enzymes that degrade the polysaccharide components of the microalgal cell wall, facilitating the release of intracellular proteins (Fig. 5). Compared to conventional extraction methods, EAE offers a more selective, environmentally sustainable, and efficient approach to protein recovery [67]. The process typically involves three key stages: pretreatment of algal biomass, enzymatic hydrolysis of the cell wall, and separation and purification of the extracted proteins. The effectiveness of EAE depends on enzyme selection, reaction conditions, and the composition of the microalgal cell wall [66]. The EAE process consists of sequential steps designed to enhance enzyme accessibility and optimize protein recovery. The first stage involves pretreating the algal biomass to disrupt the structural integrity of the cell wall and improve enzyme penetration [93]. Pretreatment methods include milling, sonication, heat treatment, or solvent exposure, each of which enhances enzyme access to the intracellular matrix. Following pretreatment, the biomass is incubated with specific cell wall-degrading enzymes that selectively degrade polysaccharides reinforcing the microalgal cell wall, facilitating intracellular protein release. The most commonly used enzymes in both laboratory and industrial applications include cellulases, hemicellulases, pectinases, and proteases [94]. After enzymatic hydrolysis, the mixture undergoes separation processes such as centrifugation, filtration, or ultrafiltration to isolate the extracted proteins from residual biomass. The protein-rich extract is then subjected to downstream purification to enhance protein concentration and quality [95]. The efficiency of enzymatic hydrolysis is highly dependent on the cell wall composition of the specific microalgal species, necessitating customized enzymatic treatments for optimal results. Additionally, key process parameters—including enzyme selection, hydrolysis time, pH, and temperature—must be optimized to maximize protein recovery rates [96].

EAE offers several advantages over traditional protein extraction methods, making it an attractive choice for sustainable and high-yield protein recovery [67]. Enzymes efficiently degrade complex polysaccharides in the microalgal cell wall, enhancing the release and recovery of proteins. This process improves overall protein yield compared to mechanical or chemical disruption methods. Moreover, enzyme selection can be tailored to target specific polysaccharide components, reducing protein degradation and minimizing contamination with unwanted compounds, thereby ensuring higher purity of the extracted proteins [97]. Unlike conventional extraction techniques that require high temperatures, harsh chemicals, or excessive energy consumption, EAE is an environmentally friendly alternative. The enzymes used in this method are biodegradable and derived from natural sources, reducing ecological impact. Additionally, EAE allows for flexible optimization of enzyme combinations, dosages, and reaction conditions to achieve desired protein yields and quality, making it highly adaptable for industrial-scale applications [98].

Several studies have explored the application of EAE for protein extraction from various microalgal species, demonstrating its versatility and efficiency. EAE has been successfully applied to extract proteins from a wide range of algae, including *Chlorella*, *Spirulina*, and *Nannochloropsis* [99, 100]. Despite its numerous advantages, EAE faces several challenges that must be addressed to enhance its commercial viability. Microalgal cell wall composition varies significantly between species, necessitating tailored enzyme formulations for efficient extraction [59]. Future research should focus on developing enzyme blends that can be applied across multiple microalgal strains. One major limitation of EAE is the high cost of industrial enzymes, which poses a challenge for large-scale applications [101]. Strategies such as enzyme immobilization, recombinant enzyme production, and cost-effective fermentation processes could enhance the economical feasibility of EAE [102]. Combining EAE with other extraction techniques, such as UAE, PEF treatment, or supercritical fluid extraction (SFE), may further improve protein yield and extraction efficiency. SFE utilizes supercritical CO₂ (often combined with water or ethanol) to disrupt cells and extract proteins, offering an environmentally friendly alternative by avoiding toxic solvents [103]. Additionally, SFE is characterized by high extraction efficiency and purity. However, its limitations include high equipment and operational costs, as well as the requirement for specialized handling [104]. Hybrid approaches could leverage the strengths of multiple techniques while mitigating individual limitations. While EAE has been successfully demonstrated in laboratory-scale studies, large-scale industrial implementation requires further process optimization, cost-benefit analyses, and advancements in enzyme delivery methods—such as immobilized enzyme systems for continuous processing [105].

### Microwave-assisted extraction

Microwave-assisted extraction (MAE) is an advanced technique for the efficient recovery of proteins from microalgal biomass (Fig. 5). This method utilizes microwave irradiation at 2.45 GHz to induce dielectric heating, which occurs when polar solvents and water within microalgae absorb microwave energy [106]. The dielectric heating mechanism causes rapid molecular vibration, increasing intracellular temperature. As a result, water evaporation generates pressure within the cells, leading to cell wall disruption and enhanced protein release. Additionally, MAE disrupts hydrogen bonds and induces the movement of dissolved ions, further enhancing solvent penetration into the algal matrix. Compared to conventional extraction techniques, MAE offers several advantages, including faster processing times, improved extraction efficiency, higher protein yields, and reduced solvent consumption, making it an environmentally sustainable approach for microalgal protein extraction [107].

The efficiency of MAE is influenced by several key parameters, including microwave power, temperature, processing time, solvent-to-sample ratio, algal species, and pretreatment conditions [108]. These factors play a critical role in determining protein yield and extract purity. The general mechanism of MAE for microalgal protein extraction involves dielectric heating, intracellular pressure buildup, and enhanced mass transfer [109]. Microwaves induce oscillations in polar molecules (e.g., water and proteins), generating frictional heating that facilitates the disruption of algal cell walls. The rapid intracellular temperature increase leads to localized water evaporation, creating pressure that enhances protein release [61]. Additionally, the movement of dissolved ions and disruption of hydrogen bonds promote solvent diffusion into the microalgal biomass, improving protein extraction efficiency. Due to its unidirectional heat and mass transfer characteristics, MAE minimizes thermal degradation of proteins, thereby preserving the purity and functionality of extracted biomolecules [110].

Several studies have investigated the effectiveness of MAE in microalgal protein recovery, highlighting its potential for industrial applications. For instance, Mahali and Sibi [111] examined MAE for protein extraction from *Arthrospira platensis*, utilizing microwave irradiation at 2.45 GHz for 3 min, with a radiation power of 1 kW and a liquid-to-solid ratio of 15 mL/5.0 g Dw. Their findings indicated that MAE achieved an impressive 78% protein yield. Additionally, protein solubility was significantly influenced by pH, with maximum solubility (74.9%) observed at pH 9.0, while the lowest solubility (0.27%) was recorded at pH 5.0. This study underscores the efficiency of MAE for microalgal protein recovery and highlights the critical role of pH in optimizing protein solubility.

MAE offers several advantages over conventional extraction techniques, making it a promising method for microalgal protein recovery. One of its key benefits is its ability to significantly reduce extraction time compared to traditional thermal and mechanical methods. The application of high microwave power enables rapid heating, which accelerates cell wall disruption and protein release [112]. Additionally, microwave energy enhances protein solubilization, leading to higher recovery rates [107]. Unlike conventional heat-based extraction methods, MAE minimizes thermal degradation, thereby preserving the structural integrity and functional properties of extracted proteins [92]. MAE also offers environmental benefits, as it requires lower solvent volumes, reducing waste generation and overall environmental impact. Furthermore, its reduced energy consumption makes it a more sustainable alternative to conventional extraction techniques. Another advantage of MAE is its versatility, as it can be applied to various microalgal species and biomass types, making it suitable for industrial-scale protein extraction [113]. Additionally, process parameters such as temperature, power, and solvent composition can be easily adjusted and optimized to maximize extraction efficiency for different microalgal strains.

Despite its advantages, MAE presents certain challenges that must be addressed for large-scale commercial applications. The efficiency of MAE varies depending on microalgal species, biomass composition, and solvent selection [114]. Additionally, high microwave power or prolonged exposure times can result in excessive heating, potentially compromising protein stability [115]. Although MAE has been extensively studied at the laboratory scale, scaling up remains challenging due to the difficulty of achieving uniform microwave distribution in large biomass volumes. Developing continuous-flow microwave systems may improve scalability and industrial applicability. Integrating MAE with UAE, EAE, or PEF treatment could further enhance protein recovery rates while reducing processing time and energy consumption. However, the high initial investment required for microwave systems may limit widespread adoption in the microalgae industry [116]. Further research on cost-benefit analysis and process optimization is needed to enhance economical feasibility and facilitate industrial-scale implementation of MAE.

Despite advancements in extraction methods, several challenges remain: Advanced techniques such as PEF and SFE require high initial investment, limiting their widespread adoption. Additionally, microalgal species exhibit diverse cell wall compositions, necessitating method optimization for each strain. While EAE and SFE are environmentally friendly, further cost reductions are required to ensure their viability at an industrial scale. To address these challenges, integrating multiple extraction techniques—such as enzyme pretreatment combined with UAE—can maximize protein yield and improve efficiency. Furthermore, biorefinery approaches, which involve extracting multiple high-value compounds (e.g., proteins, lipids, and pigments), are crucial for enhancing economical feasibility. In addition, advancements in synthetic biology—such as engineering microalgae strains with weakened cell walls—could further facilitate protein extraction and improve overall process efficiency.

Recent advances in microalgal protein extraction offer promising solutions to enhance efficiency, sustainability, and scalability. While conventional methods remain widely used, innovative techniques such as EAE, UAE, and PEF are making significant progress in optimizing protein yield while minimizing environmental impact [82]. The future of microalgal protein extraction lies in the integration of emerging technologies with biorefinery concepts, creating a more sustainable and economically viable approach. These advancements are paving the way for more efficient and eco-friendly production of microalgal proteins, which hold great potential as alternative protein sources across various industries.

## Digestibility and bioavailability of microalgal proteins

Digestibility is a crucial parameter for evaluating the bioavailability of nutrients in microalgae-derived proteins, particularly for human consumption and aquaculture applications [117]. Bioavailability encompasses a series of post-consumption processes, including protein digestibility, solubility in the gastrointestinal tract, absorption into the circulatory system, and eventual assimilation [118]. High digestibility enhances the absorption of essential amino acids and bioactive peptides, which are broken down by human digestive proteases, thereby improving the nutritional value of microalgae-based proteins [119]. Assessing the digestibility of microalgal proteins under gastric and intestinal conditions is essential to determine their suitability as alternative protein sources in human and animal diets. Digestibility assessments can be conducted using simulated gastrointestinal digestion models, which replicate enzymatic hydrolysis in the stomach and intestines [120]. However, comprehensive in vivo studies remain necessary to validate the bioavailability and nutritional efficacy of microalgae-derived proteins.

Microalgae have attracted attention as sustainable protein sources in aquaculture due to their high nutritional value, functional bioactive compounds, and well-balanced amino acid profiles. In this context, Sarker et al. [121] investigated the potential of microalgae-based feeds by combining two commercially available species: *Nannochloropsis oculata*, a residual biomass obtained after oil extraction for nutraceuticals, and docosahexaenoic acid (DHA)-rich *Schizochytrium.* The study aimed to develop an advanced fish-free diet for Nile tilapia as a replacement for conventional fish oil-based diets. The microalgae-based feed demonstrated superior performance, with significant improvements in growth parameters, weight gain, and feed conversion ratio compared to standard fish-based diets. Additionally, it resulted in enhanced fillet lipid, DHA, and protein content, indicating improved nutritional quality, and superior in vitro protein digestibility, suggesting greater hydrolysis and absorption potential. Despite a slightly higher feed cost ($0.68/kg vs. $0.64/kg for the reference feed), the microalgae-based formulation exhibited a lower economic conversion ratio ($0.95/kg tilapia vs. $1.03/kg for the standard diet), highlighting its cost-effectiveness [121]. These findings underscore the potential of microalgae-derived proteins as nutritionally beneficial and economically viable alternatives to fishmeal and fish oil in aquaculture.

The digestibility of microalgal proteins has been assessed using simulated gastrointestinal digestion models, which provide insights into their enzymatic hydrolysis and potential bioavailability. Kazir et al. [119] investigated protein digestibility in two marine macroalgae species, *Ulva* and *Gracilaria*. After two hours of simulated digestion, the study found that *Gracilaria* protein hydrolysis reached 68.1% at the end of the gastric phase, indicating efficient breakdown by pepsin, while *Ulva* protein hydrolysis was 47.8%, suggesting lower pepsin susceptibility. Following the intestinal phase, complete protein hydrolysis was observed for *Gracilaria*, whereas *Ulva* proteins reached 89.4% hydrolysis, demonstrating the effectiveness of chymotrypsin and trypsin in further protein breakdown. These findings suggest that microalgal proteins are highly digestible by human and animal digestive enzymes, potentially enhancing their absorption and bioavailability in the intestine.

The high digestibility of microalgal proteins positions them as promising alternatives to conventional protein sources for both human nutrition and aquaculture. However, several factors must be considered to optimize their application. Variations in cell wall composition and protein structure influence enzymatic hydrolysis efficiency, necessitating further research to refine processing techniques such as enzymatic hydrolysis, PEF treatment, and MAE to enhance protein digestibility [76]. Additionally, microalgal proteins may contain bioactive peptides with health benefits, including antioxidant, anti-inflammatory, and immunomodulatory properties [122]. Future studies should explore the potential health-promoting effects of microalgae-derived protein hydrolysates. While microalgae-based feeds have demonstrated high nutritional efficiency, cost reductions through biorefinery approaches and industrial optimization are essential for widespread adoption. Integrating microalgal proteins into plant-based diets could further enhance the nutritional profile of vegan and vegetarian food products [123].

Microalgal proteins exhibit high digestibility and bioavailability, making them valuable for both human nutrition and aquafeed applications. In vitro and in vivo studies have demonstrated efficient hydrolysis by digestive enzymes, indicating strong potential for absorption and assimilation [124, 125]. The success of microalgae-based diets in aquaculture further underscores their economical viability and sustainability. Future research should focus on optimizing extraction methods, evaluating bioactive properties, and scaling up production to facilitate the global adoption of microalgae-derived proteins as a sustainable alternative to conventional protein sources.

## Life cycle assessment, environmental impact, and economical viability of microalgal protein production

The global food industry is undergoing a significant transformation as the demand for sustainable and alternative protein sources continues to rise. Microalgae have garnered considerable attention as a viable protein alternative due to their high protein content, rapid biomass accumulation, and ability to thrive in non-arable land and wastewater environments. These characteristics position them as a promising solution to address the environmental and economical challenges associated with traditional protein sources, such as land degradation, water overuse, and GHG emissions.

**Fig. 6:**
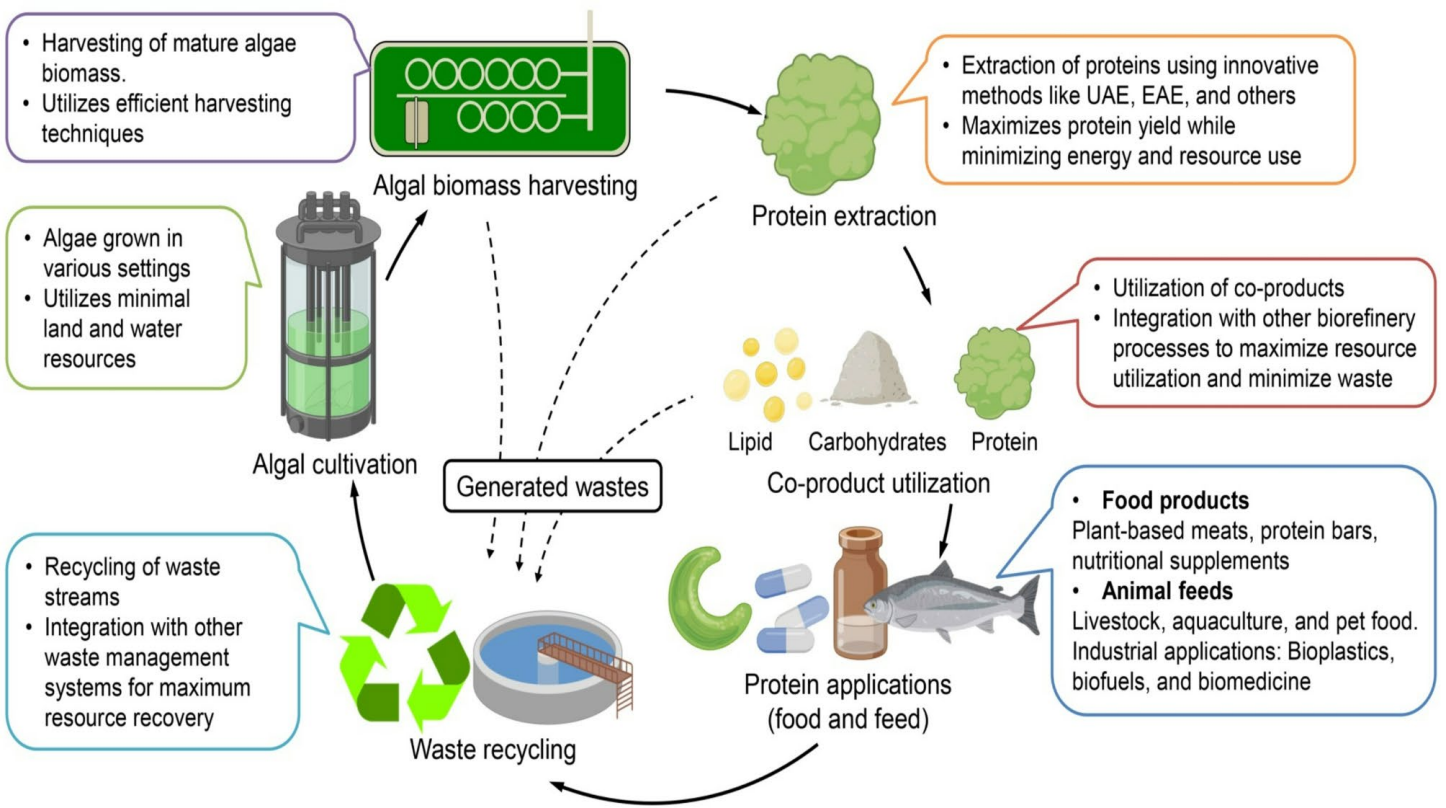
Schematic representation of the microalgal protein production process, emphasizing a circular economy approach. The diagram illustrates key steps, including algal cultivation, biomass harvesting, protein extraction, co-product utilization, and protein applications. It highlights the integration of waste recycling for resource recovery and the generation of food, feed, and industrial products, underlining the sustainability of the process through life cycle assessment (LCA) principles

A LCA is crucial to evaluate the sustainability of microalgal protein production, taking into account factors such as resource utilization, environmental footprint, and economical feasibility [126]. Despite their potential benefits, challenges like high energy consumption, production costs, and contamination risks remain significant barriers to large-scale commercialization. This section provides an in-depth analysis of LCA in microalgal protein production, examining its environmental impact and economical viability while comparing it with conventional protein sources. Figure 6 presents a comprehensive framework for microalgal protein production, highlighting a circular economy approach and the integration of LCA principles to enhance environmental sustainability and economical viability. The figure outlines the holistic process of microalgal protein production, with a focus on sustainability achieved through the application of LCA and circular economy methodologies. It showcases the potential of microalgae as a versatile, eco-friendly, and economically viable resource for diverse applications, aligning with global objectives for sustainable development [127].

### Life cycle assessment of microalgal protein production

The key stages of the LCA for microalgal protein production include: cultivation, harvesting and dewatering, extraction, and waste management. Microalgae can be cultivated using either open raceway ponds or closed PBRs, each with distinct environmental and economical implications. Open raceway pond systems are cost-effective and require minimal infrastructure. However, they are associated with lower productivity, high evaporation rates, and contamination risks [128]. The lower biomass yield often necessitates larger cultivation areas, raising concerns about land use. In contrast, PBRs offer higher productivity and greater control over factors such as light exposure, temperature, and nutrient supply. Nevertheless, they are energy-intensive, primarily due to artificial lighting and temperature regulation, which significantly increases production costs and environmental impact [31]. Compared to traditional protein sources like soy or beef, microalgae cultivation has a significantly smaller land footprint. Unlike agricultural proteins, microalgae do not compete for arable land, thereby reducing deforestation and habitat loss [129]. Additionally, microalgae act as carbon sinks by sequestering CO₂ from industrial emissions, offering potential benefits for climate change mitigation [130].

Harvesting and dewatering represent one of the most energy-intensive stages in microalgal protein production. Various harvesting methods include flocculation (aggregation of algal cells), centrifugation (highly efficient but energy-intensive), and membrane filtration (lower energy requirement but higher initial cost). Water use efficiency is a critical sustainability parameter. While microalgae require significantly less water than soy or livestock protein production, evaporation losses in open pond systems can pose a challenge [128]. On the other hand, microalgal protein extraction involves mechanical, enzymatic, and solvent-based methods, each with distinct environmental trade-offs. Mechanical disruption techniques, such as bead milling and high-pressure homogenization, break down algal cell walls, enhancing protein bioavailability [60]. Enzymatic hydrolysis, which uses enzymes to degrade cell walls, is effective but costly. Solvent extraction is efficient in isolating proteins but can generate chemical waste. Although protein extraction is relatively efficient compared to animal protein production, further process optimization is needed to reduce energy consumption and minimize chemical waste [131]. Sustainable microalgal protein production can be enhanced through waste valorization. Residual biomass from protein extraction can be repurposed for biofuels (energy production), animal feed (as a cost-effective alternative to fishmeal and soy protein), biofertilizers (to enrich soil fertility), and nutraceuticals (such as antioxidants, carotenoids, and omega-3 fatty acids). By adopting a circular economy approach, the overall environmental footprint and economical feasibility of microalgal protein production can be significantly improved [132].

#### Feasibility analysis using LCA

LCA studies have identified key factors influencing the feasibility of microalgal protein production, including energy use, water footprint, land use, and GHG emissions. In this context, the cultivation phase, particularly in PBRs, is the most energy-intensive, largely due to the demands of lighting and temperature control [10]. In terms of water footprint, microalgae require significantly less water compared to traditional crops like soybeans, positioning them as a more sustainable option in terms of water consumption [133]. Regarding land use, microalgae can be cultivated on non-arable land, minimizing competition with food crops and reducing pressure on fertile agricultural areas [7]. When it comes to GHG emissions, microalgal protein production can achieve a lower carbon footprint than conventional animal protein production, provided it is powered by renewable energy and incorporates CO₂ recycling [134]. Table 4 provides a comparative analysis of microalgal protein and conventional protein sources [16, 128, 135].

**Table 4:** Advantages of microalgal protein over conventional proteins

Parameter	Microalgal protein	Soy protein	Whey protein	Beef protein
Protein yield (%)	40–70%	35–50%	60–90%	15–25%
Production cost ($/kg)^*^	5–15	1–2	4–8	5–20
Land use efficiency	High	Moderate	Low	Very low
Water use (L/kg protein)^*^	Low (~ 500)	Moderate (~ 2500)	High (~ 4500)	Very high (~ 15,000)
Greenhouse gas emissions (kg CO₂-eq/kg protein)^*^	Low (~ 2–5)	Moderate (~ 3–6)	High (~ 10)	Very high (~ 50)
References	[135]	[135]	[128]	[16]

^*^Specific numerical values for production costs, water use, and greenhouse gas emissions are subject to variability based on production methods and regional factors

### Environmental impact of microalgal protein production

Microalgal protein production offers several environmental advantages over conventional protein sources but also faces certain sustainability challenges. Among its benefits, microalgae require significantly less land compared to traditional protein sources, using 95% less land than livestock and 60% less land than soy protein [117, 136]. Unlike soybean cultivation and animal agriculture, microalgae can be grown in saline water, wastewater, or industrial effluents, reducing reliance on freshwater resources. Additionally, microalgae absorb atmospheric CO₂ and can be integrated with industrial carbon capture systems, contributing to the mitigation of GHG emissions [134]. However, microalgal protein production is not without its drawbacks. Some production methods, particularly those involving energy-intensive photobioreactors, have a global warming potential four times higher than that of soy protein [137]. Furthermore, certain microalgal species have the potential to accumulate heavy metals and contaminants, raising potential health concerns [138].

### Economical viability of microalgal protein production

Microalgae hold significant economical potential across various industries, including food, nutraceuticals, cosmetics, and biofuels. However, high production costs remain a major challenge. Among the economical advantages, microalgae exhibit high biomass productivity, capable of doubling their biomass within 24 h, enabling rapid large-scale protein production [139]. Another advantage is the generation of co-product revenue, as microalgal processing yields valuable by-products such as lipids, pigments, and antioxidants, creating multiple revenue streams [140]. Furthermore, microalgal cultivation can be integrated with wastewater treatment and carbon capture systems, enhancing overall profitability. Despite these benefits, economical challenges persist. For instance, the cost of microalgal protein is 5–10 times higher than that of soy protein [31]. To address this, cost reduction strategies are essential, including innovations in photobioreactor design, CO₂ injection techniques, and automation to improve efficiency and lower expenses.

Overall, LCA studies indicate that microalgal protein production has a lower environmental footprint compared to animal-based proteins. However, it is currently more expensive than traditional plant-based proteins [16]. Advances in cultivation technologies, energy efficiency, and economies of scale are expected to reduce costs, potentially making microalgal protein a competitive alternative in the future. Although current production costs exceed those of conventional plant-based proteins, ongoing research and technological innovations are likely to improve the economical feasibility of microalgal proteins, positioning them as a sustainable and viable option in the global protein market.

## Challenges and future prospects in microalgal protein production

Despite significant advancements, several challenges must be addressed to improve the scalability, economical feasibility, and sustainability of microalgal protein production. The cost of producing microalgal protein remains significantly higher than that of soy and other plant-based proteins [117]. Factors such as expensive bioreactors, energy-intensive cultivation, and costly harvesting techniques limit large-scale commercial production. Cost reduction strategies include advancements in low-cost photobioreactor designs and open pond systems, the utilization of industrial CO₂ waste streams to enhance microalgal growth, and the implementation of automated and AI-driven monitoring systems to optimize cultivation efficiency [141, 142].

Harvesting and dewatering microalgal biomass account for 20–30% of total production costs [143]. Conventional methods such as centrifugation and filtration are energy-intensive and require further optimization. Improving harvesting efficiency could be achieved through the development of bioflocculants and autoflocculating microalgae strains [144], as well as the integration of membrane filtration and electroflocculation technologies to reduce energy consumption [145]. Another major challenge is the rigid cell walls of many microalgal species, which hinder protein extraction efficiency. While mechanical, enzymatic, and chemical extraction methods are available, each has limitations in cost, efficiency, and scalability. Hybrid extraction techniques, such as ultrasound-assisted enzymatic extraction, could enhance protein yield, while microbial fermentation and pre-treatment strategies may improve cell wall digestibility, facilitating more efficient protein extraction [146].

Some microalgal proteins exhibit low digestibility and bioavailability, which can impact their nutritional value. Cell wall components such as chitin and fiber may interfere with protein absorption. To address these challenges, genetic engineering approaches are needed to enhance essential amino acid profiles and improve protein solubility [147]. Additionally, the development of enzyme-assisted processing techniques is essential for breaking down indigestible compounds in microalgal proteins [148]. While microalgae require less land and water than traditional crops, photobioreactor-based systems can have high carbon footprints due to energy-intensive operations. Utilizing renewable energy sources (e.g., solar and wind) can help reduce the environmental impact of cultivation facilities [149]. Additionally, the adoption of wastewater-based microalgal cultivation can reduce freshwater demand and improve sustainability, while enhancing CO₂ sequestration efficiency to offset industrial carbon emissions [150].

The lack of regulatory approvals in some regions limits the integration of microalgal proteins into mainstream food markets [17]. Furthermore, consumer acceptance of microalgae-based foods remains low due to concerns about taste, texture, and lack of awareness. To address these issues, it is crucial to develop standardized regulatory guidelines for microalgal protein safety and labeling, expand public awareness campaigns that highlight the health and environmental benefits of microalgal proteins, and introduce food processing innovations (e.g., flavor enhancement and texturization) to improve the sensory appeal of microalgal protein products.

## Conclusion

Microalgae provide a nutrient-rich, sustainable, and scalable protein source that can play a crucial role in addressing global food security challenges. Their high protein content, rapid growth rates, and ability to thrive in non-arable land make them a promising alternative to conventional livestock and crop-based proteins. Recent advancements in cultivation techniques and extraction methods—including PEF, UAE, EAE, and MAE—have significantly improved protein yields and extraction efficiency. Additionally, LCAs indicate that microalgal protein production has a lower environmental footprint than animal-based proteins. However, further process optimizations are needed to reduce energy costs and enhance economical feasibility. Challenges such as high production costs, harvesting inefficiencies, and regulatory barriers must be addressed through innovations in biorefinery models, genetic engineering, and sustainable production strategies. Integrating renewable energy sources, CO₂ sequestration technologies, and waste valorization approaches can make microalgal proteins cost-competitive and environmentally friendly for human nutrition and animal feed applications. With continued research, policy support, and industrial investment, microalgal protein production has the potential to transform the global protein market, contributing to a more sustainable and resilient food system.

## Data Availability

No datasets were generated or analysed during the current study.
